# The multifaceted role of extracellular vesicles in prostate cancer-a review

**DOI:** 10.20517/cdr.2023.17

**Published:** 2023-07-28

**Authors:** Divya Prakash Jain, Yirivinti Hayagreeva Dinakar, Hitesh Kumar, Rupshee Jain, Vikas Jain

**Affiliations:** ^1^Department of Pharmaceutics, JSS College of Pharmacy, JSS Academy of Higher Education & Research, Mysuru 570015, India.; ^2^Department of Pharmaceutical Chemistry, JSS College of Pharmacy, JSS Academy of Higher Education & Research, Mysuru 570015, India.

**Keywords:** Extracellular vesicles, prostate cancer, stemness, chemoresistance, therapeutic delivery, diagnosis

## Abstract

Prostate cancer is the second most prominent form of cancer in men and confers the highest mortality after lung cancer. The term “extracellular vesicles” refers to minute endosomal-derived membrane microvesicles and it was demonstrated that extracellular vesicles affect the environment in which tumors originate. Extracellular vesicles’ involvement is also established in the development of drug resistance, angiogenesis, stemness, and radioresistance in various cancers including prostate cancer. Extracellular vesicles influence the general environment, processes, and growth of prostate cancer and can be a potential area that offers a significant lead in prostate cancer therapy. In this review, we have elaborated on the multifaceted role of extracellular vesicles in various processes involved in the development of prostate cancer, and their multitude of applications in the diagnosis and treatment of prostate cancer through the encapsulation of various bioactives.

## INTRODUCTION

One of the most common forms of cancer identified in men is prostate cancer (PCa), which is also one of the deadliest, the second most common type of cancer in men after lung cancer, and the fifth most prevalent mortality cause in males^[1]^. In the United States of America alone in 2021, more than 248,000 instances of PCa were identified in men, and just under 34,000 fatalities were attributed to the disease^[2]^. Over 1.4 million cases worldwide were reported, with more than 300,000 deaths related to PCa^[3]^. Currently, multiple treatment strategies are used against PCa. These therapies include surgery, radiation therapy, active surveillance, and proton therapy. Apart from these, other strategies including chemotherapy, hormonal therapy, cryosurgery, and HIFU (high-intensity focused ultrasound) are also part of the current treatment regime based on the clinical conditions and outcomes^[4-10]^. Active surveillance emerged as the management option for the low-risk PCa and various strategies were developed for the same^[11]^. Urological guidelines proposed various treatment options depending on the stage of PCa. For example, patients with low-risk diseases are offered active surveillance and active treatment such as surgery or radiation therapy. For immediate-risk disease patients, radical prostatectomy, radiotherapeutic therapy, and pelvic lymph node dissection is the treatment option. The same treatment option applies to patients with high-risk localized disease. In addition, radiotherapy and surgery are used as a treatment for locally advanced disease^[12]^. Hormonal therapy, also known as androgen deprivation therapy (ADT), is a traditional and standard therapy against PCa for over 60 years and shows its effect through the decrease in serum testosterone^[13]^. Hormonal therapies have also shown combinative strategies with other standard treatment options such as radiotherapy, non-invasive treatments through medications, and hormonal therapy with surgery^[14,15]^. Hormonal therapy with drugs in prostate cancer provides effective tumor growth and progression control, boosting patient outcomes and quality of life. At present, commonly used medications in hormonal therapy involve drugs such as enzalutamide, abiraterone, goserelin, bicalutamide, etc^[16-21]^.

Following hormonal therapy for a while, the tumor develops into metastatic castration-resistant PCa (mCRPC), which has a low rate of survival and few treatment options^[22-24]^. The manner in which multilaminar bodies, particularly extracellular vesicles, influence the general environment, the processes, and the expansion of PCa cells is one of the areas that has demonstrated a significant amount of potential when it comes to the treatment of PCa.

**Figure 1:**
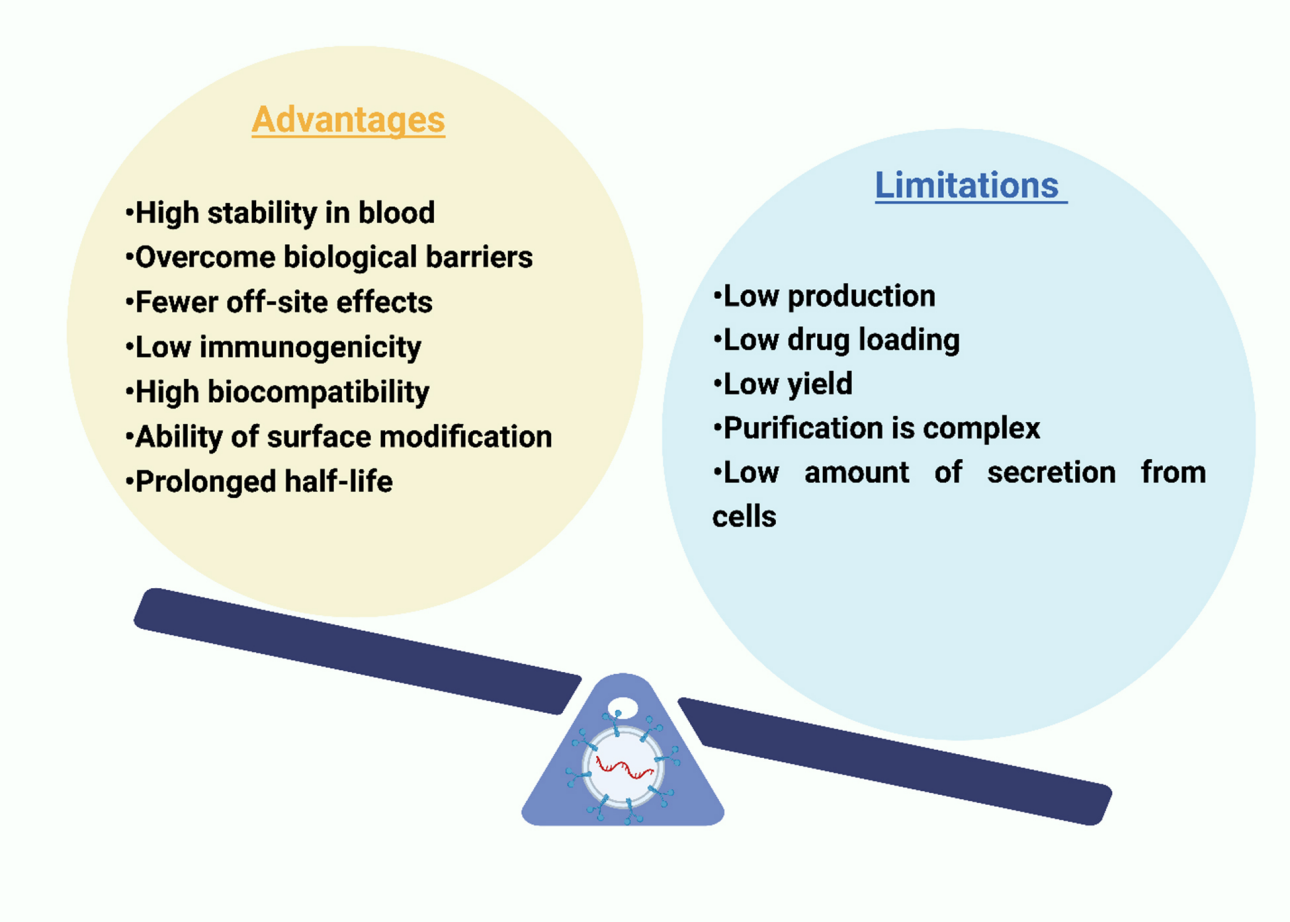
Graphical representation of advantages and limitations of extracellular vesicles.

The term “extracellular vesicles” was primarily used by Rose Johnstone and her colleagues in 1970 to refer to minute endosomal-derived membrane microvesicles^[25,26]^. Extracellular vesicles offer several advantages, such as the ease of therapeutic cargo loading (drugs, siRNA), the ability to penetrate through biological barriers, low immunogenicity, ease of cellular uptake, ease of surface modification, etc^[27]^. However, limitations such as difficulty in obtaining high quantities of pure extracellular vesicles, purification process, and disadvantages pertaining to various isolation techniques limit the application of extracellular vesicles in drug delivery. The advantages and disadvantages associated with extracellular vesicles are graphically depicted in Figure 1^[28]^.

Substantial numbers of researchers concentrated their attention on the roles of extracellular vesicles in the act of tumors. There is a lot of interest in how extracellular vesicles affect the environment in which tumors originate, the role that extracellular vesicles play in the progression of drug resistance in cancer cells in response to anti-cancer medications, and the role that extracellular vesicles can play in mitigating the effects of cancer and perhaps halting the process of cancer growth as well as their potentially intricate role in the delivery of chemotherapeutic agents and other bioactives in the tumor cells. Extracellular vesicles, which initially were considered molecular waste bins, now have shown a multifaceted role in cancer. Extracellular vesicles serve a significant function in angiogenesis through the transport of various pro-angiogenic biomolecules, such as vascular endothelial growth factor (VEGF), microRNAs, and matrix metalloproteinases (MMPs), and also act as key agents in metastasis through their involvement in restructuring metastatic sites to support cancer cell colonization^[29-31]^; Extracellular vesicles have a key role as biomarkers in the cancer development through its components including proteins, nucleic acids and also from other biofluids [Table 1]^[32]^. While talking about the exosomal role in PCa, they have been indulged in acting as the key agent in the progression of PCa through numerous mechanisms including changes in the tumor microenvironment and angiogenesis, through the metastasis and cell proliferation, and in the drug resistance in PCa tumor^[33-43]^.

**Table 1:** List of biomarkers from extracellular vesicles in prostate cancer

**Biomarkers from extracellular vesicles**		**The role played in prostate cancer**	**Cell line/ model used**	**References**
Proteins	HSP70	Provides drug resistance, promotes invasion, stemness, and metastasis	PC3 and LNCaP	^[44-46]^
	Caveolin 1 (CAV1)	Progression and metastasis of Prostate cancer; inhibit apoptosis in prostate cancer cells.	LNCaP and PC3 cell lines	^[47,48]^
	Integrin alpha-2 (ITGA2)	Mediates cancer progression and metastasis; possible role in alteration of AR phenotype and development of aggressive prostate cancer	CRPC-derived extracellular vesicles, LNCaP cell lines	^[49,50]^
	Annexin A2	Enhances the release of IL-6, promoting the proliferation of prostate cancer; migration and adhesion to osteoblasts	DU145, LNCaP, PC-3	^[51-53]^
	Vimentin	Associated with invasion and metastasis via Src regulation	PC-3M-1E8 PC-3M-2B4	^[54]^
RNA	miR-21	Promotes growth as well as proliferation following the surgical castration; Also promotes invasion and apoptosis resistance	LNCaP, androgen-dependent PC-3 cell lines, and DU-145 cell lines	^[55,56]^
	TMPRSS2-ERG fusion	Promotes cell proliferation	TMPRSS2-ERG-positive PCa xenograft models	^[57]^
	miRNA-221/222	Promotes oncogenesis and progression of prostate cancer through p27(Kip1) downregulation	PC-3(aggressive prostate carcinoma model), LNCaP, and 22Rv1 cell line models	^[58]^
	MALAT1	Through sponging miR-145 promotes cell proliferation, migration, and invasion	LNCaP and CWR22Rv1 cell lines	^[59]^
	HOTAIR (HOX transcript antisense RNA)	Decreasing the inhibitory effect of hepaCAM on MAPK signaling promotes invasion and metastasis.	Samples collected from patients at Dept. of Urology, First Affiliated Hospital of Chongqing Medical University, China	^[60]^

In this review article, we have described the multifaceted functions that extracellular vesicles play in PCa. This review focuses on the pro-tumorigenic role of extracellular vesicles in the key processes engaged in the development of PCa, such as stemness, chemoresistance, radioresistance, angiogenesis, and metastasis. Furthermore, the application of extracellular vesicles in the diagnosis and delivery of therapeutic moieties such as the siRNA, drugs, and phytoconstituents are also elaborated in this review.

## PRO-TUMORIGENIC ROLE OF EXTRACELLULAR VESICLES IN PCa

### Role of extracellular vesicles in stemness of PCa

Stemness refers to the phenomenon that defines the cells' capability of self-renewal and differentiation^[61]^. It is a characteristic ability shown by adult stem cells to proliferate, and bolster their new generation of daughter cells and also interact with their environment to maintain a balance between quiescence, proliferation, and regeneration^[62]^. In the case of cancer stem cells (CSCs), this phenomenon acts as a malignant equivalent to normal stem cells. Apart from the common features such as maintenance, sustainability, supporting the microenvironment, and self-renewal, the essential differences found in the cancer stem cells refer to extra features that include heterogeneity in the cell population, high resistance towards hostile factors like quiescence, chemotherapeutic agents, hypoxia and low nutrient levels^[62-67]^.

Stemness plays an influential role in the development of PCa. The prostate stem cells reside in the basal and luminal layers and are a major target for the oncogenic transformation^,^ which suggests a role in the genesis of PCa^[68]^. CSCs demonstrated an altered gene expression in exposure to hypoxia, nutrient deficiency, and oxidative stress, rendering them more mobile, invasive, and resilient to further stress. CSCs are anticipated to have endured epithelial-mesenchymal transition + , and the transition to mesenchymal marker expression is frequently one measurement of PCa progression. CSCs are predicted to invade locally and then metastasize^[69]^.

Extracellular vesicles portray a substantial part in the stemness of PCa. They have been shown to be involved in multiple features exhibited by PCa stem cells, such as resistance against hypoxia and tumor progression, promotion of epithelial-to-mesenchymal transition (EMT), and also the transformation of other stem cells into cancer stem cells. Ramteke *et al.* in their work discovered that extracellular vesicles derived from hypoxic PCa cells promote the cancer-associated fibroblast (CAF) phenotype in prostate stromal cells, and also elevate the property of stemness and protruding of naive PCA (PCa) cells. They subjected human PCA PC3 and LNCaP cells to normoxic and hypoxic conditions, respectively, and extracted the extracellular vesicles released in both situations. They observed increased amounts of Annexin II, heat shock proteins (HSP90 and HSP70), and tetraspanins (CD63 and CD81) in hypoxic extracellular vesicles, which led to LNCaP and PC3 cell invasiveness and motility. Aside from that, they discover an increased amount of metalloproteins as well as increased levels of various signaling molecules. Further, in proteome analysis, they found an increased number of proteins in hypoxic extracellular vesicles, which promotes epithelial adheres junction pathway remodeling and proteins, especially in naïve PC3 cells. This study overall suggests how extracellular vesicles promote the stemness of PCa cells, affecting significant features such as invasiveness and tumor microenvironment, leading to aggressive PCa^[44]^. In another study, it was found how extracellular vesicles containing PSGR (Prostate-specific G-protein coupled receptor) promote the migration, invasiveness, and stemness of low-aggressive PCa cells. They used transcriptome sequencing to determine the differentially expressed (DE) mRNAs in low invasive cells incubated with overly expressed PC3 extracellular vesicles or negative control (NC) extracellular vesicles. They also discovered that the PSGR was stably overexpressed in PC3 cells. Internalization of PC3 PSGR + extracellular vesicles in LNCaP and RWPE-1 cells greatly promoted cell migration and invasion. After PC3 PSGR + exosome incubation, E-cadherin expression declined, while vimentin, Snail, SOX2, and OCT4a expression elevated in low invasive cells. This resulted in findings indicating that extracellular vesicles released via PCa cells induce invasiveness and stemness^[70]^. An interesting study was published talking about how extracellular vesicles derived from PCa cells promoted the neoplastic reprogramming of adipose stem cells derived from the patient. They identified that extracellular vesicles obtained from PCa facilitated the transformation of adipose stem cells into neoplastic cells in the patient due to changes in the cell microenvironment. They observed that pASCs (PCa patients derived adipose-stem cells) primed with PCa cell conditioned media (CM) produced prostate-like neoplastic lesions in vivo and replicated aggressive tumors in secondary recipients, in contrast to normal ASCs (adipose-stem cells). The cytogenetic aberrations and mesenchymal-to-epithelial transition of the pASC tumors, along with the expression of epithelial, neoplastic, and vasculogenic markers, were evocative of molecular features of PCa tumor xenografts. This suggests that extracellular vesicles play a tremendous role in not only promoting the stemness, invasiveness, and growth of PCa cells but can also be an agent advocating the transformation of other stem cells into oncogenic cells^[71-73]^.

### Extracellular vesicles in drug resistance and radioresistance of PCa

#### Extracellular vesicles and drug resistance

The development of resistance to treatment drugs has been an extremely difficult obstacle to overcome in PCa treatment^[43,74]^. The use of chemotherapy is still considered to be one of the more traditional methods for treating advanced PCa^[5]^. However, it has been demonstrated that several variables, including the heterogeneity of the tumor, epigenetic control by miRNAs, and the combinatorial outcomes of various signaling pathways, including NF-κB/IL-6, Hedgehog, mTOR (mammalian target of rapamycin), Akt/PI3K, MAPK/ERK, and somatostatin receptors, all of these elements result in drug resistance in tumor cells^[75-79]^.

According to numerous research, extracellular vesicles are involved in the development of medication resistance in PCa^[42,74,80]^. Extracellular vesicles have been discovered as the mechanism underlying resistance against the drug enzalutamide, and in PCa, the emergence of therapy-induced neuroendocrine differentiation stages, as demonstrated by Bhagirath D *et al.* in their research^[74]^. In addition, it was observed that BRN2 and BRN4, which are neural transcription factors, were liberated in PCa extracellular vesicles after the treatment with enzalutamide. These transcription factors are essential for the neuroendocrine remodeling of prostate adenocarcinomas^[74,81,82]^. Kharaziha *et al.* discovered with the usage of nanoparticle tracking analysis that a greater quantity of extracellular vesicles released by DU145 PCa cells docetaxel-resistant than by DU145 PCa docetaxel-sensitive cells^[80]^. Extracellular vesicles were shown to be responsible for resistance against docetaxel in docetaxel-susceptible PCa cells (DU145, LNCaP, and 22Rv1)^[42]^. Following administration of the cells with docetaxel-resistant extracellular vesicles versions of 22Rv1 and DU145 (22Rv1RD and DU145RD, respectively), authors observed the liberation of MDR-1/Pgp, which is a multidrug resistance protein 1/P-glycoprotein and this transporter protein engaged in the efflux of a wide range of exogenous objects, including antineoplastic medications, from extracellular vesicles, played a probable role in imparting resistance to docetaxel-susceptible cells^[42]^. Another study looked through an interesting pathway to study the chemoresistance in PCa cells. In the study, exosome-derived miR-27a, which portrays a substantial role in chemoresistance in cells of PCa, was examined. When administered with doxorubicin, cisplatin, and docetaxel in PCa cells, there was a tremendous spike in the levels of the miR-27a; they also co-treated PC3 cells (PCa cells) with primary prostate fibroblasts (PSC27 cells) to analyze tumor treatment resistance mechanisms. The results additionally demonstrate that exosome-derived miR-27a produced by PSC-27 cells enhanced chemoresistance by suppressing P53 gene expression^[35]^. Although it is not the commonly observed pathway for drug resistance, Saari *et al.* in their research made an interesting discovery. They observed that using two contrasting populations of extracellular vesicles (microvesicle- and exosome-enriched) as paclitaxel carriers in autologous PCa increased the cytotoxicity effect of the paclitaxel, regardless of the fact that cancer cell viability increased without the vesicles, but the overall net cytotoxicity effect remained increased. They also observed that this phenomenon was irrespective of the EV population and cell lines tested^[83]^*.*

In recent years, various studies demonstrated the role played by the extracellular vesicles in the development of drug resistance in PCa. Shan *et al.* observed that miRNA-423-5p from extracellular vesicles, which was secreted from cancer-associated fibroblasts, was promoting chemoresistance in prostate cancer. They observed that miRNA-423-5p from extracellular vesicles promoted chemoresistance through inhibition of GREM2 via the TGF-β pathway for taxane derivatives while suppressing the TGF-β pathway was able to partially reverse the chemoresistance^[84]^. In another study, Kato *et al.* utilized the serum extracellular vesicles containing CD44v8-10 mRNA as the diagnostic marker for docetaxel resistance in prostate cancer. The results showed that the levels of CD44v8-10 protein and mRNA in cell lysates and extracellular vesicles were higher in PC-3R cells, which was a docetaxel-resistant cell line compared to normal PC-3 cells. This showed that CD44v8-10 protein had a role in the development of chemoresistance^[85]^. The role of syntaxin-6-mediated extracellular vesicles in the regulation of enzalutamide resistance in prostate cancer was demonstrated. The authors observed that the enzalutamide-resistant cell lines (CWR-R1, C4-2B, and LNCaP) had a higher amount of extracellular vesicle secretion (about 2-4) times than the enzalutamide-sensitive cells. The observed mechanism underlying was found to be the upregulation of syntaxin-6 accompanied by the increase in colocalization with CD-63 in enzalutamide resistance cell lines. They also observed that knocking down syntaxin-6 with siRNAs resulted in a reduction in cell count and an enhancement in cell death in the presence of enzalutamide^[86]^.

#### Extracellular vesicles and radioresistance

Apart from the drug resistance shown in PCa in the above section, extracellular vesicles are also associated with radioresistance in PCa^[87]^. In the case of PCa, following the radiation therapy, increased levels of HSP72-containing extracellular vesicles were found in PCa, leading to the conclusion that HSP72-containing extracellular vesicles are a possible patron, leading to pro-inflammatory cytokine production and immune modulation^[88]^. Other than this, another phenomenon was observed in the cancer stem cells that tend to show more resistance against radiation therapy. This allows the production of extracellular vesicles that will deliver the resistance phenotype to recipient cells^[87]^. This will allow for the formation of more resistant cancer cells to radiation therapy. Regarding PCa, research has demonstrated the presence of extracellular vesicles released from prostate stem cells; these vesicles have the capacity for autophagy and can also modulate the sensitivity towards radiation therapy^[89,90]^. All of this suggests a strong negative role of extracellular vesicles not only as a key agent against drug and chemoresistance, but extracellular vesicles also play a quintessential role in the emergence of resistance against radiation therapy in PCa^[91]^. Radiation therapy, still one of the mainstream therapies for the treatment of cancer across all types, can affect the release of content from extracellular vesicles, which has already been affected by the composition and abundance of the extracellular vesicles, affecting the extracellular vesicle-based intracellular communication.

### Extracellular vesicles in metastasis of PCa

Aside from their influence at metastatic locations, tumor-derived extracellular vesicles have been shown to have a role in the invasion, development, and metastasis of tumors by interacting with cells at isolated, pre-metastatic organ locations^[92,93]^. This transpires through the establishment of tumor-nurturing microenvironments, a technique known as “pre-metastatic niche generation”. The formation requires protracted intercellular communication facilitated by soluble or membrane-bound proteins originating from the original tumor^[94-96]^.

The tumor-originated extracellular vesicles are responsible for the extracellular matrix (ECM) remodeling via the accumulation of fibronectin and the promotion of crosslinking by the ECM-modifying enzyme lysyl oxidase (LOX). This is done with the intention of improving bone marrow-derived cell adherence, which is a critical component of the pre-metastatic niche. To improve the adherence of bone-marrow-derived cells, tumor-originated extracellular vesicles remodel the ECM through the aggregation of fibronectin and crosslinking ECM^[97-99]^.

Intriguingly, new research conducted by Henrich *et al.* indicates convincingly that homeostasis of cholesterol in bone marrow myeloid cells is the mediator of communication between bone marrow and PCa cells through extracellular vesicles. They observed that bone marrow myeloid cells absorb PCa extracellular vesicles, leading to an activated NF-κB signaling, improved osteoclast formation *in vitro* and *in vivo*, and lessened myeloid thrombospondin-1 expression^[100]^. A tailored biomimetic strategy involving myeloid cells *in vitro* and *in vivo* lowering cholesterol levels prevented PCa EV uptake by recipient myeloid cells, eliminated NF-κB activity, maintained thrombospondin-1 expression, decreased osteoclast differentiation, and yielded a 77% reduction in metastatic burden^[100]^. During early stages of metastasis, the EMT is a pivotal critical step. This state is triggered by tumor-derived extracellular vesicles’ autocrine and paracrine signaling and targeting proteins such as transforming growth factor-beta (TGF-β) and catenin, which are EMT-related proteins^[101,102]^. Extracellular vesicles derived from PCa DU145 and PC3 cells generate a type of TGF- β capable of initiating fibroblast-myofibroblast transformation by activating the TGF-SMAD signaling pathway. This TGF-β is also capable of promoting tumorigenesis and inhibiting immune response^[103,104]^. McAtee *et al.* showed in their research that prostate tumor cells containing extracellular vesicles have a protein called hyaluronidase 1 (Hyal1) in them. This protein encourages the relocation of prostate stromal cells, which ultimately speeds up the evolution of PCa^[105]^. Research conducted by Josson *et al.* showed that the microRNA miR-409, which has a crucial part in the EMT in PCa, was observed in stromal-derived extracellular vesicles^[106]^. Furthermore, integrins are known to play a role in metastasis and progression of cancer, and in PCa, the α_v_β_6_ integrin is shown to enhance the ability to migrate^[107]^. Furthermore, the integrins derived from extracellular vesicles are also known to play a role in angiogenesis^[108]^. In a research study, the proteins Integrin Subunit Alpha 3 (ITGA3) and Integrin Subunit Beta 1 (ITGB1) were found in extracellular vesicles recovered from LNCaP and PC3 cells, released in the urine of PCa patients, both of them contributing to the diaspora and invasion of both tumors^[109]^. It is worth noting that Elamgeed *et al.* reported that exosome-derived miR-130b, miR-125b, and miR-155 promote mesenchymal-epithelial transition (MET) and neoplastic transformation and stem cells isolated from PCa patients’ adipose tissue^[71]^. Honeywell *et al.* illustrated in their research how miR-105 obtained from tumor-derived extracellular vesicles serves as a tumor suppressor and targets the cyclin-dependent kinase 6 (CDK6), which suppresses the cell proliferation^[110]^. Another interesting route was discovered, bolstering a pathway for metastasis in PCa. It was also demonstrated that androgen receptors were expressed by PCa cells-derived extracellular vesicles, which, after nuclear localization, led to enhanced cell proliferation^[111]^. Metastasis of lymph nodes to distant organs can be observed through the probable mediation of extracellular vesicles. Even though a mystery is shrouded suggesting the initiation of this process by which tumor cells, it is possible that lymph node metastasis could occur through extracellular vesicles. The authors of the research, Maolake *et al.*, revealed that the tumor necrosis factor-α (TNF-α) provides a huge contribution towards metastasizing PCa lymph nodes using LNCaP, DU145, LNCaP-SF, and PC3 cell lines of PCa can be seen through the activation of chemokine (C-C motif) ligand 21/CC chemokine receptor 7 (CCL21/CCR7) axis and this discovery may mediate the influence that extracellular vesicles have on metastasis through the lymphatic pathway^[112]^. Furthermore, in another research conducted, it was discovered that from the PC3, DU145, CWR-R1 PCa, and LNCaP cells, which were derived from designated metastatic PCa cell lines, integrin alpha 2 subunits (ITGA2) were found packaged in extracellular vesicles. The fact that inhibiting the release of extracellular vesicles to recipient cells in these metastatic PCa cells by knocking down ITGA2 revealed a possible function for ITGA2 in contributing to the development of the illness and the aggressive phenotypes found in PCa. With an elevated Gleason Score of 9, lymph node metastatic tissues were found to have higher ITGA2 expression than lower Gleason Score of 7 PCa tissues, implying that ITGA2 may play a role in enhancing lymph node metastasis^[49]^. Furthermore, no notable differences were observed in ITGA2 protein expression levels between 24 primary PCa tissues and their matched metastatic lymph node tissues. Regardless of the fact that there were no key differences, this was the case.

### Role in progression and angiogenesis

Extracellular vesicles are released constitutively by tumor cells of diverse sources. These extracellular vesicles play essential roles in the transformation of tumors into malignant forms and the progression of cancers. Extracellular vesicles’ effects are mediated by the transfer of cargo, which includes a range of proteins as well as RNA (including miRNAs) and DNA^[113,114]^. Cancer cells essentially require to sustain their attributes, such as immortality, continued proliferative activity, evasion of immune reactions, angiogenesis, continued invasion and metastasis, and resistance to cell death or apoptosis. The tumor microenvironment supplies this medium, which includes tumor stromal cells such as mesenchymal stromal cells, fibroblasts, pericytes, and immune cells such as T & B lymphocytes. They serve as the growth medium for the tumor cells, which are located within the tumor. The transformation of normal stroma cells into reactive stroma cells, which promotes the growth of cancer cells as well as metastasis, is a feature that is distinctive of the progression of cancer. After being impacted, the stromal cells will employ extracellular vesicles to regulate the microenvironment of the tumor, therefore promoting both the development of the tumor and its ability to metastasize^[115-117]^. TME extracellular vesicles now perform the role of cellular communicators in addition to assisting with a range of activities. In the instance of PCa, DeRita *et al.* discovered that protein Src was found in PCa cell extracellular vesicles. Through integrin activation, this protein activates focal adhesion kinase, which leads to angiogenesis and metastasis^[34]^. Eventually, tumor cells and the stromal cells that are in close vicinity will engage in a persistent kind of interaction^[115]^. By encouraging the differentiation of fibroblasts into a myofibroblast-like phenotype that produces angiogenesis and tumor development, cancer extracellular vesicles that contain TGF- β play critical roles in the generation of stroma that promotes the growth of tumors^[30-118]^. The TGF-β/SMAD3 cascade has to be activated to produce this effect. The extracellular vesicles that are produced by PCa cells have a high level of latent TGF-β expression. Through focal adhesion kinase via integrin activation, this protein triggers metastasis and angiogenesis^[119,120]^.

**Figure 2:**
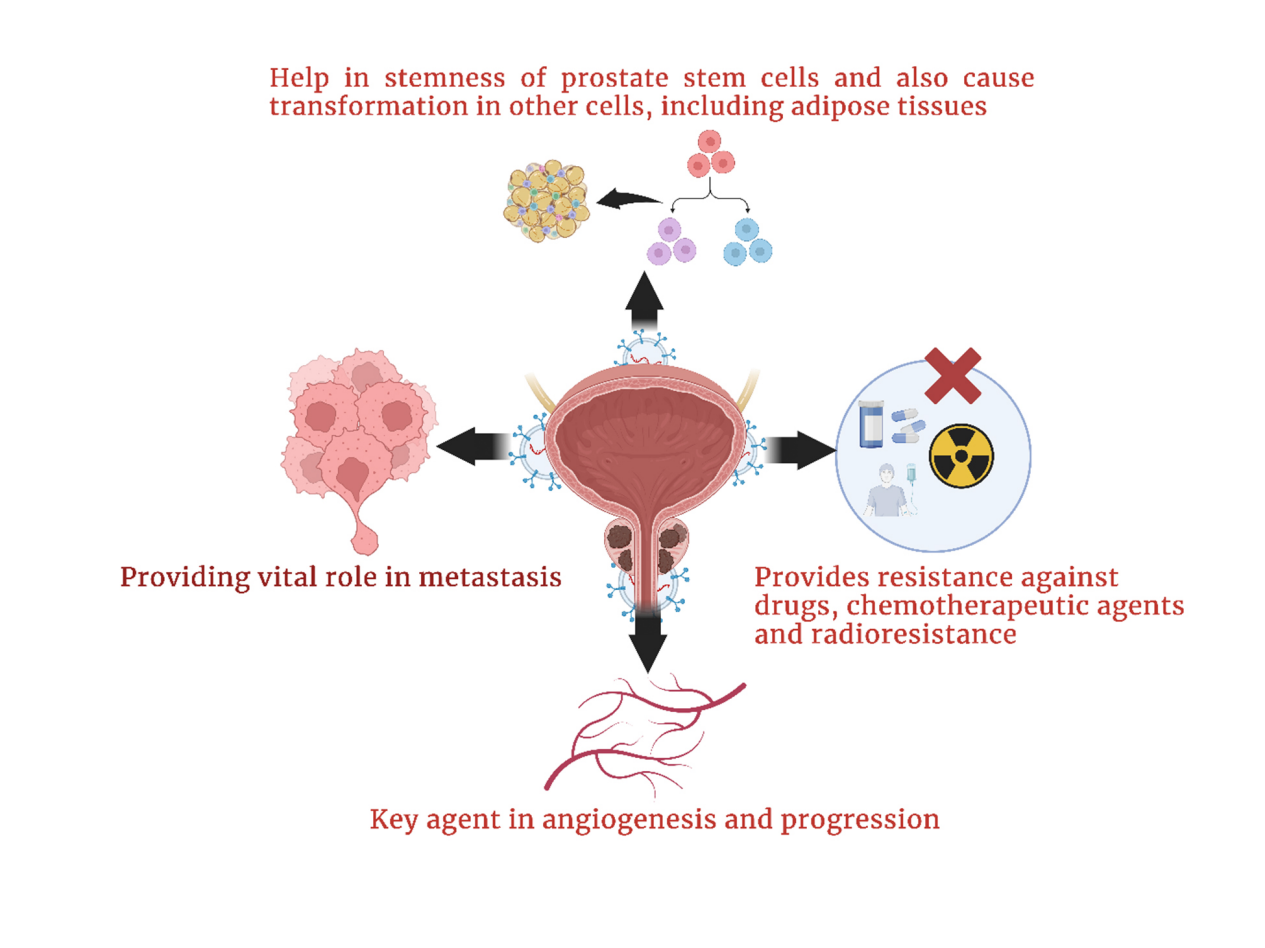
Graphical representation of the pro-tumorigenic characteristics of extracellular vesicles in PCa.

The production of extracellular vesicles by PCa cells enables the delivery of sphingomyelin and CD147 into endothelial cells, which facilitates cancer cell migration and endothelial cell pro-angiogenic activity^[121-122]^. Proteins such as c-Src tyrosine kinase, IGF-R (insulin-like growth factor 1 receptor) and FAK (focal adhesion kinase) all play imperative roles in the formation and advancement of prostate tumors^[6,15,34,123-125]^. Extracellular vesicles derived from PCa exhibit significant concentrations of these proteins. Angiogenesis is stimulated by the cross-talk that occurs between Src and IGF-1R^[34,126]^. Extracellular vesicles rich in Src have been shown to have the ability to stimulate angiogenesis in animal models that have prostate tumors. This is because Src is overexpressed within plasma extracellular vesicles of mice with prostate tumors. Furthermore, Src and IGF1-R affect angiogenesis by activating VEGF and VEGF-C, respectively^[127,128]^. This is the case for both of these and focal adhesion kinase, which, if activated, leads to metastasis and angiogenesis^[34,129]^. Cancer extracellular vesicles that contain TGF-β play critical roles in the formation of stroma that is favorable to the development of tumors. These extracellular vesicles encourage the differentiation of fibroblasts into a myofibroblast-like phenotype, which in turn stimulates angiogenesis and tumor expansion^[130-131]^. The activation of the TGF-β/SMAD3 cascade is the mechanism by which this impact is produced^[132]^. PCa cells are responsible for producing extracellular vesicles that have a high level of latent TGF-β expression. This latent TGF-β adheres to the exosome surface via proteoglycans, promoting the activation of SMAD3-dependent signaling cascades^[133,134]^. Figure 2 shows a graphical representation of pro-tumorigenic characteristics of extracellular vesicles in PCa.

## THE ROLE OF EXTRACELLULAR VESICLES IN THE DIAGNOSIS AND THERAPY OF PCa

### Utilization of extracellular vesicles in the of PCa diagnosis-recent advances

On the positive side, extracellular vesicles are widely studied for the diagnosis and therapy of PCa^[135,136]^. The extracellular vesicles derived from the urine, plasma, and semen^[40,137,138]^ have been utilized for the diagnosis of PCa. Several studies demonstrated the utilization of extracellular vesicles in PCa diagnosis^[39,139-141]^. A recent clinical investigation revealed that PCa patients may be discriminated from both Benign Prostate Hyperplasia (BPH) and healthy subjects by the expression of PSA on plasmatic extracellular vesicles. In a study, it was demonstrated as a new strategy for differentiating not just PCa from healthy persons but also from benign hypertrophy^[142]^. In another study, it was found that extracellular vesicles from individuals with PCa exhibited overexpression of CA IX levels, which is related to intraluminal pH, as compared to healthy persons, and demonstrated that the PCa extracellular vesicles are acidic in nature and can be used as a biomarker in PCa^[143]^. Since the literature depicts the use of extracellular vesicles in the diagnosis of PCa, in this review, we will focus on the latest advances in extracellular vesicles for the diagnosis of PCa. Li *et al.* developed a superparamagnetic conjunctions and molecular beacons (SMC-MB) based platform in which it detected and captured the prostate-specific membrane antigen-positive extracellular vesicles, thereby depicting its efficiency in the diagnosis of PCa^[144]^.

In another study, an economical, easy, and non-invasive method was developed for diagnosing PCa in which the extracellular vesicles containing surface proteins and miRNAs from extracellular vesicles were detected at the same time and permitted the analysis of particular miRNAs and surface proteins through one reaction^[145]^. A nanoplatform for specific and quick detection, which consists of off-on signal responses and reversible conjunction, was developed in which high-affinity particles of Fe_3_O_4_@SiO_2_@TiO_2_ were employed for exosome capture and selectivity was improved through the fluorescence response PMSA aptasensor which aid in the tumor exosome detection. The study concluded that this method was fruitful for quick diagnosis^[146]^. In another study reported, a microfluidic Raman biochip with immunoassay was developed for the separation and analysis of extracellular vesicles. This chip was able to differentiate the samples of PCa patients and normal samples. Furthermore, this system detected the extracellular vesicles in one hour and thus can be explored as the detection test for PCa^[147]^. In summary, here we discussed some of the recent advances in the utilization of extracellular vesicles for the diagnosis of PCa. To the best of our knowledge, extracellular vesicles have been extensively utilized for the detection of PCa, and various advances such as the Raman chip and others have also been developed and employed for much rapid, non-invasive and sensitive analysis of the samples. Soon, various additional options will be developed to enhance diagnosis using extracellular vesicles.

### Extracellular vesicles in the PCa therapy

**Figure 3:**
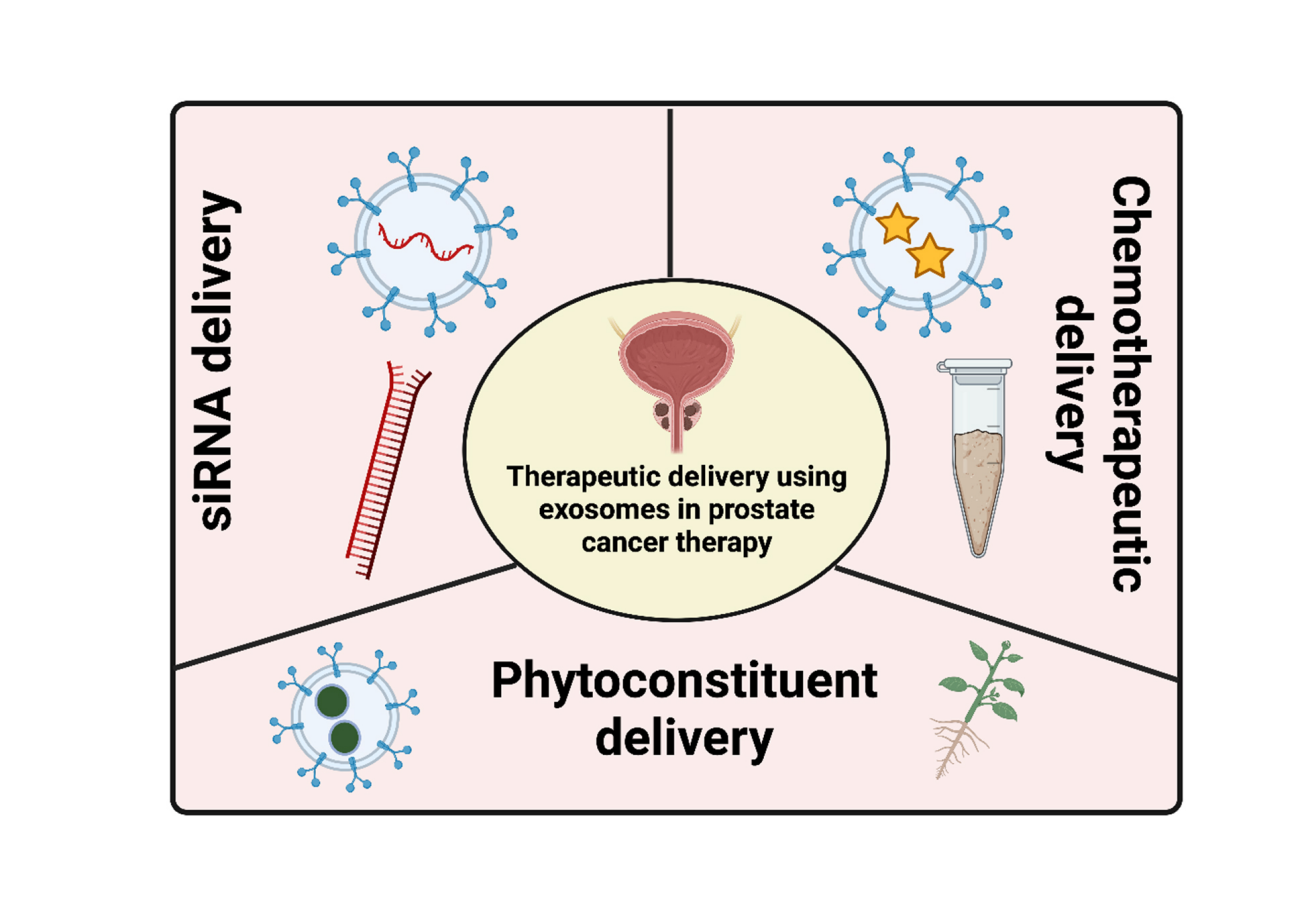
The application of extracellular vesicles in the therapy PCa.

Another such advantage is its application in drug delivery. Owing to their advantages over conventional delivery systems, such as their high ability to overcome biological barriers, enhanced stability, better targeting, unnecessary accumulation in the liver, and low toxicity, extracellular vesicles are widely utilized for the delivery of drugs as well as other therapeutic moieties such as siRNA, phytoconstituents^[28,148]^. The extracellular vesicles are obtained from various cells, such as tumor cells, immune cells, and mesenchymal stem cells, and are also derived from bovine milk. Of these, the extracellular vesicles derived from mesenchymal stem cells and tumor cells are widely explored for therapeutic purposes^[149]^. In this section, the role of extracellular vesicles in the delivery of various therapeutic moieties such as the siRNA, chemotherapeutics, and phytoconstituents are discussed [Figure 3].

#### siRNA delivery through extracellular vesicles

It was shown that due to the underlying mechanisms, extracellular vesicles are found to be the right candidates for the delivery of siRNA^[148]^. For this reason, extracellular vesicles are utilized for the successful delivery of siRNA for targeting various cancers such as breast cancer^[150]^, colon cancer, gastric cancer, and others, including PCa^[151,152]^. In the context of PCa, a research study was aimed to determine the efficiency of siRNA in the inhibition of SIRT6 in a PCa model. It was demonstrated that the siRNA via the engineered extracellular vesicles caused the downregulation of the SIRT6 and blocked the metastasis and tumor growth^[153]^. In another study by Krishn *et al.*, it was demonstrated that delivery of siRNAs targeting ITGB6 into the PCa cells (PC3) led to the downregulation of β6 subunit expression and also led to cell adhesion and migration of PCa cells on a specific substrate of αVβ6^[154]^. In an *in vivo* study on mice bearing subcutaneous prostate carcinoma, the extracellular vesicles modified with polyethyleneimine showed strong inhibition of tumor growth, which is due to the significant knockdown of the target gene^[155]^. Even though siRNA delivery through extracellular vesicles has been researched widely in recent times, in our opinion, the extracellular vesicles can be utilized to a greater extent for the targeted therapy of PCa due to their effectiveness in inhibiting the target genes as well as the advantages it offers.

#### Drug delivery through extracellular vesicles

The extracellular vesicles are also used to carry drugs to the delivery site [Figure 3]. Followed by its isolation, the drugs are conjugated with them, which can reduce the toxicity and biocompatibility issues^[156]^. Saari *et al.* isolated the extracellular vesicles from PCa cells (PC-3 and LNCaP) through a process called differential centrifugation. An increased cytotoxic effect was observed when the paclitaxel was loaded into the extracellular vesicles derived from autologous PCa cells. Furthermore, endocytosis was the key pathway involved in the delivery of paclitaxel to the parental cells, thereby demonstrating the effectiveness of extracellular vesicles as carriers for drug delivery^[83]^. In another study, the urinary-derived exosomal system containing doxorubicin Exo-PMA/Fe-HSA@DOX nano vectors was explored for synergistic chemodynamic/low-dose chemotherapy of PCa. The nanosystem was shown to cause substantial internalization *in vitro* and to suppress the EGFR/AKT/NF-B pathways in cells^[157]^. Not just drugs, but a combination of both drugs and siRNAs have also been delivered for PCa therapy. For example, folate-conjugated extracellular vesicles (Co-Exo-FA) were developed which are obtained from nano-complex loaded macrophages. Docetaxel and PLK1 siRNA were loaded in this system. It was shown that the system blocked the PLK1 gene in addition to its effect on tumor growth and reduced toxicity, which demonstrated the synergistic effect of drug and siRNA combination against castrate-resistance PCa^[158]^.

#### Phytoconstituent delivery through extracellular vesicles

It has been reported that extracellular vesicles aid in the delivery of phytochemicals across biological barriers and have also been used against cancer^[159]^. However, in PCa, there are very few phytochemicals delivered through the exosome in our knowledge. Overall, extracellular vesicles are utilized to transport various molecules to PCa patients, and still, a lot of molecules such as various siRNAs, drugs, and phytochemicals can be loaded into extracellular vesicles for the targeted inhibition of various genes associated with PCa, tumor growth and to target the various steps involving in the emergence of PCa. In our opinion, this area remains much unexplored.

## CONCLUSION AND FUTURE PERSPECTIVE

Extracellular vesicles show a significant role when it comes to the entire scenario involving PCa. Once considered garbage bags of cells, extracellular vesicles are now being researched for their multifarious roles in cancer development. In the case of PCa, they have shown a dual persona: a negative aspect enables extracellular vesicles to contribute to proliferation and metastases while conferring resistance against the majority of chemotherapeutic agents and therapies, including radiation therapy. However, they also demonstrate a beneficial side, serving as diagnostic biomarkers and delivery vesicles for a vast array of agents, including nucleic acids, diagnostic agents, pharmaceuticals, and many more. This review aims to comprehensively cover both aspects of extracellular vesicles in PCa, encompassing their negative roles, such as involvement in angiogenesis, metastasis, proliferation, stemness, and resistance to multiple agents, alongside their roles as diagnostic agents and delivery agents for various pharmaceuticals.

There is a promising future for extracellular vesicles in treating PCa. While extracellular vesicles have shown tremendous progress as delivery vesicles for multiple agents, there is a limited number of studies focused on the delivery of phytochemicals in PCa using extracellular vesicles. Additionally, further studies can be conducted to explore how their role in drug resistance can be utilized positively while delivering antineoplastic agents in PCa. PCa has been a major threat in the male population, but future studies and the utilization of extracellular vesicles are expected to play a significant role in its treatment and mitigating its effects worldwide.

## DECLARATIONS

### Copyright

© The Author(s) 2023.

## References

[1] Rawla P (2019). Epidemiology of prostate cancer. World J Oncol.

[2] Siegel RL, Miller KD, Fuchs HE, Jemal A (2021). Cancer statistics, 2021. CA Cancer J Clin.

[3] Wang L, Lu B, He M, Wang Y, Wang Z, Du L (2022). Prostate cancer incidence and mortality: global status and temporal trends in 89 countries from 2000 to 2019. Front Public Health.

[4] Chen FZ, Zhao XK (2013). Prostate cancer: current treatment and prevention strategies. Iran Red Crescent Med J.

[5] Nader R, El Amm J, Aragon-Ching JB (2018). Role of chemotherapy in prostate cancer. Asian J Androl.

[6] Rodríguez SA, Arias Fúnez F, Bueno Bravo C (2014). Cryotherapy for primary treatment of prostate cancer: intermediate term results of a prospective study from a single institution. Prostate Cancer.

[7] Alkhorayef M, Mahmoud MZ, Alzimami KS, Sulieman A, Fagiri MA (2015). High-intensity focused ultrasound (HIFU) in localized prostate cancer treatment. Pol J Radiol.

[8] Poon DMC, Wu S, Ho L, Cheung KY, Yu B (2022). Proton therapy for prostate cancer: challenges and opportunities. Cancers.

[9] Desai K, McManus JM, Sharifi N (2021). Hormonal therapy for prostate cancer. Endocr Rev.

[10] Kim EH, Bullock AD (2018). Surgical management for prostate cancer. Mo Med.

[11] Walker CH, Marchetti KA, Singhal U, Morgan TM (2022). Active surveillance for prostate cancer: selection criteria, guidelines, and outcomes. World J Urol.

[12] Mottet N, van den Bergh RCN, Briers E (2021). EAU-EANM-ESTRO-ESUR-SIOG guidelines on prostate cancer-2020 update. Part 1: screening, diagnosis, and local treatment with curative intent. Eur Urol.

[13] Gomella LG, Singh J, Lallas C, Trabulsi EJ (2010). Hormone therapy in the management of prostate cancer: evidence-based approaches. Ther Adv Urol.

[14] Laverdière J, Gomez JL, Cusan L (1997). Beneficial effect of combination hormonal therapy administered prior and following external beam radiation therapy in localized prostate cancer. Int J Radiat Oncol Biol Phys.

[15] Recine F, Sternberg CN (2015). Hormonal therapy and chemotherapy in hormone-naive and castration resistant prostate cancer. Transl Androl Urol.

[16] Bolla M, Gonzalez D, Warde P (1997). Improved survival in patients with locally advanced prostate cancer treated with radiotherapy and goserelin. N Engl J Med.

[17] Anderson J, Al-Ali G, Wirth M (2013). Degarelix versus goserelin (+ antiandrogen flare protection) in the relief of lower urinary tract symptoms secondary to prostate cancer: results from a phase IIIb study (NCT00831233). Urol Int.

[18] Fizazi K, Tran N, Fein L, LATITUDE Investigators (2017). Abiraterone plus prednisone in metastatic, castration-sensitive prostate cancer. N Engl J Med.

[19] Thakur A, Roy A, Ghosh A, Chhabra M, Banerjee S (2018). Abiraterone acetate in the treatment of prostate cancer. Biomed Pharmacother.

[20] Beer TM, Armstrong AJ, Rathkopf DE, PREVAIL Investigators (2014). Enzalutamide in metastatic prostate cancer before chemotherapy. N Engl J Med.

[21] OH WK (2002). Secondary hormonal therapies in the treatment of prostate cancer. Urology.

[22] Sartor AO (2011). Progression of metastatic castrate-resistant prostate cancer: impact of therapeutic intervention in the post-docetaxel space. J Hematol Oncol.

[23] Karantanos T, Corn PG, Thompson TC (2013). Prostate cancer progression after androgen deprivation therapy: mechanisms of castrate resistance and novel therapeutic approaches. Oncogene.

[24] Dong L, Zieren RC, Xue W, de Reijke TM, Pienta KJ (2019). Metastatic prostate cancer remains incurable, why?. Asian J Urol.

[25] Johnstone RM (2005). Revisiting the road to the discovery of exosomes. Blood Cells Mol Dis.

[26] Ha D, Yang N, Nadithe V (2016). Exosomes as therapeutic drug carriers and delivery vehicles across biological membranes: current perspectives and future challenges. Acta Pharm Sin B.

[27] Peng H, Ji W, Zhao R (2020). Exosome: a significant nano-scale drug delivery carrier. J Mater Chem B.

[28] Jiang XC, Gao JQ (2017). Exosomes as novel bio-carriers for gene and drug delivery. Int J Pharm.

[29] H Rashed M, Bayraktar E, K Helal G (2017). Exosomes: from garbage bins to promising therapeutic targets. Int J Mol Sci.

[30] Olejarz W, Kubiak-Tomaszewska G, Chrzanowska A, Lorenc T (2020). Exosomes in angiogenesis and anti-angiogenic therapy in cancers. Int J Mol Sci.

[31] Adem B, Vieira PF, Melo SA (2020). Decoding the biology of exosomes in metastasis. Trends Cancer.

[32] Sumrin A, Moazzam S, Khan AA (2018). Exosomes as biomarker of cancer. Braz arch biol technol.

[33] Deep G, Panigrahi GK (2015). Hypoxia-induced signaling promotes prostate cancer progression: exosomes role as messenger of hypoxic response in tumor microenvironment. Crit Rev Oncog.

[34] DeRita RM, Zerlanko B, Singh A (2017). c-Src, Insulin-like growth factor i receptor, g-protein-coupled receptor kinases and focal adhesion kinase are enriched into prostate cancer cell exosomes. J Cell Biochem.

[35] Cao Z, Xu L, Zhao S (2019). Exosome-derived miR-27a produced by PSC-27 cells contributes to prostate cancer chemoresistance through p53. Biochem Biophys Res Commun.

[36] Logozzi M, Angelini DF, Iessi E (2017). Increased PSA expression on prostate cancer exosomes in in vitro condition and in cancer patients. Cancer Lett.

[37] Tang MKS, Yue PYK, Ip PP (2018). Soluble e-cadherin promotes tumor angiogenesis and localizes to exosome surface. Nat Commun.

[38] Li T, Sun X, Chen L (2020). Exosome circ-0044516 promotes prostate cancer cell proliferation and metastasis as a potential biomarker. J Cell Biochem.

[39] Shin S, Park YH, Jung SH (2021). Urinary exosome microRNA signatures as a noninvasive prognostic biomarker for prostate cancer. NPJ Genom Med.

[40] Nilsson J, Skog J, Nordstrand A (2009). Prostate cancer-derived urine exosomes: a novel approach to biomarkers for prostate cancer. Br J Cancer.

[41] Li Q, Hu J, Shi Y (2021). Exosomal lncAY927529 enhances prostate cancer cell proliferation and invasion through regulating bone microenvironment. Cell Cycle.

[42] Corcoran C, Rani S, O'Brien K (2012). Docetaxel-resistance in prostate cancer: evaluating associated phenotypic changes and potential for resistance transfer via exosomes. PLoS One.

[43] Mostafazadeh M, Samadi N, Kahroba H, Baradaran B, Haiaty S, Nouri M (2021). Potential roles and prognostic significance of exosomes in cancer drug resistance. Cell Biosci.

[44] Ramteke A, Ting H, Agarwal C (2015). Exosomes secreted under hypoxia enhance invasiveness and stemness of prostate cancer cells by targeting adherens junction molecules. Mol Carcinog.

[45] Gibbons N, Watson R, Coffey R, Brady H, Fitzpatrick J Heat-shock proteins inhibit induction of prostate cancer cell apoptosis. Prostate.

[46] Teng Y, Ngoka L, Mei Y, Lesoon L, Cowell JK (2012). HSP90 and HSP70 proteins are essential for stabilization and activation of WASF3 metastasis-promoting protein. J Biol Chem.

[47] Ariotti N, Wu Y, Okano S (2021). An inverted CAV1 (caveolin 1) topology defines novel autophagy-dependent exosome secretion from prostate cancer cells. Autophagy.

[48] Karam JA, Lotan Y, Roehrborn CG, Ashfaq R, Karakiewicz PI, Shariat SF (2007). Caveolin-1 overexpression is associated with aggressive prostate cancer recurrence. Prostate.

[49] Gaballa R, Ali HEA, Mahmoud MO (2020). Exosomes-mediated transfer of itga2 promotes migration and invasion of prostate cancer cells by inducing epithelial-mesenchymal transition. Cancers.

[50] Westendorf JJ, Hoeppner L (2007). Type I collagen receptor ({alpha}2{beta}1) signaling promotes the growth of human prostate cancer cells within the bone. Urol Oncol-Semin Ori.

[51] Tan SH, Young D, Chen Y (2021). Prognostic features of Annexin A2 expression in prostate cancer. Pathology.

[52] Shiozawa Y, Havens AM, Jung Y (2008). Annexin II/annexin II receptor axis regulates adhesion, migration, homing, and growth of prostate cancer. J Cell Biochem.

[53] Inokuchi J, Narula N, Yee DS (2009). Annexin A2 positively contributes to the malignant phenotype and secretion of IL-6 in DU145 prostate cancer cells. Int J Cancer.

[54] Ma D, Wei J, Xu G (2008). Overexpression of vimentin contributes to prostate cancer invasion and metastasis via src regulation. Anticancer Res.

[55] Li T, Li D, Sha J, Sun P, Huang Y (2009). MicroRNA-21 directly targets MARCKS and promotes apoptosis resistance and invasion in prostate cancer cells. Biochem Biophys Res Commun.

[56] Ribas J, Lupold SE (2010). The transcriptional regulation of miR-21, its multiple transcripts, and their implication in prostate cancer. Cell Cycle.

[57] Zhou F, Gao S, Han D (2019). TMPRSS2-ERG activates NO-cGMP signaling in prostate cancer cells. Oncogene.

[58] Galardi S, Mercatelli N, Giorda E (2007). MiR-221 and MiR-222 expression affects the proliferation potential of human prostate carcinoma cell lines by targeting p27Kip1. J Biol Chem.

[59] Zhang D, Fang C, Li H (2021). Long ncRNA MALAT1 promotes cell proliferation, migration, and invasion in prostate cancer via sponging miR-145. Transl Androl Urol.

[60] Li T, Liu N, Gao Y (2021). Long noncoding RNA HOTAIR regulates the invasion and metastasis of prostate cancer by targeting hepaCAM. Br J Cancer.

[61] Mushtaq M, Kovalevska L, Darekar S (2020). Cell stemness is maintained upon concurrent expression of RB and the mitochondrial ribosomal protein S18-2. Proc Natl Acad Sci U S A.

[62] Aponte PM, Caicedo A (2017). Stemness in cancer: stem cells, cancer stem cells, and their microenvironment. Stem Cells Int.

[63] Chaffer CL, Brueckmann I, Scheel C (2011). Normal and neoplastic nonstem cells can spontaneously convert to a stem-like state. Proc Natl Acad Sci U S A.

[64] Li P, Zhou C, Xu L, Xiao H (2013). Hypoxia enhances stemness of cancer stem cells in glioblastoma: an in vitro study. Int J Med Sci.

[65] Song YJ, Zhang SS, Guo XL (2013). Autophagy contributes to the survival of CD133+ liver cancer stem cells in the hypoxic and nutrient-deprived tumor microenvironment. Cancer Lett.

[66] Rao GH, Liu HM, Li BW (2013). Establishment of a human colorectal cancer cell line P6C with stem cell properties and resistance to chemotherapeutic drugs. Acta Pharmacol Sin.

[67] Chen W, Dong J, Haiech J, Kilhoffer MC, Zeniou M (2016). Cancer stem cell quiescence and plasticity as major challenges in cancer therapy. Stem Cells Int.

[68] Mei W, Lin X, Kapoor A, Gu Y, Zhao K, Tang D (2019). The contributions of prostate cancer stem cells in prostate cancer initiation and metastasis. Cancers.

[69] Harris KS, Kerr BA (2017). Prostate cancer stem cell markers drive progression, therapeutic resistance, and bone metastasis. Stem Cells Int.

[70] Li Y, Li Q, Li D (2021). Exosome carrying PSGR promotes stemness and epithelial-mesenchymal transition of low aggressive prostate cancer cells. Life Sci.

[71] Abd Elmageed ZY, Yang Y, Thomas R (2014). Neoplastic reprogramming of patient-derived adipose stem cells by prostate cancer cell-associated exosomes. Stem Cells.

[72] Cho JA, Park H, Lim EH, Lee KW (2012). Exosomes from breast cancer cells can convert adipose tissue-derived mesenchymal stem cells into myofibroblast-like cells. Int J Oncol.

[73] Gu J, Qian H, Shen L (2012). Gastric cancer exosomes trigger differentiation of umbilical cord derived mesenchymal stem cells to carcinoma-associated fibroblasts through TGF-β/Smad pathway. PLoS One.

[74] Bhagirath D, Yang TL, Tabatabai ZL (2019). BRN4 Is a novel driver of neuroendocrine differentiation in castration-resistant prostate cancer and is selectively released in extracellular vesicles with BRN2. Clin Cancer Res.

[75] Zhang A, Miao K, Sun H, Deng CX (2022). Tumor heterogeneity reshapes the tumor microenvironment to influence drug resistance. Int J Biol Sci.

[76] Zhu Y, Liu H, Xu L (2015). p21-activated kinase 1 determines stem-like phenotype and sunitinib resistance via NF-κB/IL-6 activation in renal cell carcinoma. Cell Death Dis.

[77] Coco S, Truini A, Alama A (2015). Afatinib resistance in non-small cell lung cancer involves the PI3K/AKT and MAPK/ERK signalling pathways and epithelial-to-mesenchymal transition. Target Oncol.

[78] Spetz J, Langen B, Rudqvist N (2017). Hedgehog inhibitor sonidegib potentiates^177 ^Lu-octreotate therapy of GOT1 human small intestine neuroendocrine tumors in nude mice. BMC Cancer.

[79] Margariti N, Fox SB, Bottini A, Generali D (2011). “Overcoming breast cancer drug resistance with mTOR inhibitors”. Could it be a myth or a real possibility in the short-term future?. Breast Cancer Res Treat.

[80] Kharaziha P, Chioureas D, Rutishauser D (2015). Molecular profiling of prostate cancer derived exosomes may reveal a predictive signature for response to docetaxel. Oncotarget.

[81] Quaglia F, Krishn SR, Daaboul GG (2020). Small extracellular vesicles modulated by αVβ3 integrin induce neuroendocrine differentiation in recipient cancer cells. J Extracell Vesicles.

[82] Quaglia M, Dellepiane S, Guglielmetti G, Merlotti G, Castellano G, Cantaluppi V (2020). Extracellular vesicles as mediators of cellular crosstalk between immune system and kidney graft. Front Immunol.

[83] Saari H, Lázaro-Ibáñez E, Viitala T, Vuorimaa-Laukkanen E, Siljander P, Yliperttula M (2015). Microvesicle- and exosome-mediated drug delivery enhances the cytotoxicity of Paclitaxel in autologous prostate cancer cells. J Control Release.

[84] Shan G, Gu J, Zhou D (2020). Cancer-associated fibroblast-secreted exosomal miR-423-5p promotes chemotherapy resistance in prostate cancer by targeting GREM2 through the TGF-β signaling pathway. Exp Mol Med.

[85] Kato T, Mizutani K, Kawakami K, Fujita Y, Ehara H, Ito M (2020). CD44v8-10 mRNA contained in serum exosomes as a diagnostic marker for docetaxel resistance in prostate cancer patients. Heliyon.

[86] Peak TC, Panigrahi GK, Praharaj PP (2020). Syntaxin 6-mediated exosome secretion regulates enzalutamide resistance in prostate cancer. Mol Carcinog.

[87] Ni J, Bucci J, Malouf D, Knox M, Graham P, Li Y (2019). Exosomes in cancer radioresistance. Front Oncol.

[88] Hurwitz MD, Kaur P, Nagaraja GM, Bausero MA, Manola J, Asea A (2010). Radiation therapy induces circulating serum Hsp72 in patients with prostate cancer. Radiother Oncol.

[89] Kumar D, Gupta D, Shankar S, Srivastava RK (2015). Biomolecular characterization of exosomes released from cancer stem cells: Possible implications for biomarker and treatment of cancer. Oncotarget.

[90] Li L, Liu WL, Su L, Lu ZC, He XS (2020). The role of autophagy in cancer radiotherapy. Curr Mol Pharmacol.

[91] Malla B, Zaugg K, Vassella E, Aebersold DM, Dal Pra A (2017). Exosomes and exosomal micrornas in prostate cancer radiation therapy. Int J Radiat Oncol Biol Phys.

[92] Bai S, Wang Z, Wang M (2022). Tumor-derived exosomes modulate primary site tumor metastasis. Front Cell Dev Biol.

[93] Whiteside TL Tumor-derived exosomes and their role in cancer progression. Elsevier.

[94] Liu Y, Cao X (2016). Characteristics and significance of the pre-metastatic niche. Cancer Cell.

[95] (2011). Zijl F, Krupitza G, Mikulits W. Initial steps of metastasis: cell invasion and endothelial transmigration. Mutat Res.

[96] Doglioni G, Parik S, Fendt SM (2019). Interactions in the (Pre)metastatic Niche Support Metastasis Formation. Front Oncol.

[97] Huang W, Chiquet-Ehrismann R, Orend G, Moyano J V (2001). , Garcia-Pardo A. Interference of tenascin-C with syndecan-4 binding to fibronectin blocks cell adhesion and stimulates tumor cell proliferation. Cancer Res.

[98] Høye AM, Erler JT (2016). Structural ECM components in the premetastatic and metastatic niche. Am J Physiol Cell Physiol.

[99] Peinado H, Zhang H, Matei IR (2017). Pre-metastatic niches: organ-specific homes for metastases. Nat Rev Cancer.

[100] Henrich SE, McMahon KM, Plebanek MP (2020). Prostate cancer extracellular vesicles mediate intercellular communication with bone marrow cells and promote metastasis in a cholesterol-dependent manner. J Extracell Vesicles.

[101] Xiao D, Barry S, Kmetz D (2016). Melanoma cell-derived exosomes promote epithelial-mesenchymal transition in primary melanocytes through paracrine/autocrine signaling in the tumor microenvironment. Cancer Lett.

[102] Hao Y, Baker D, Ten Dijke P (2019). TGF-β-mediated epithelial-mesenchymal transition and cancer metastasis. Int J Mol Sci.

[103] Webber J, Steadman R, Mason MD, Tabi Z, Clayton A (2010). Cancer exosomes trigger fibroblast to myofibroblast differentiation. Cancer Res.

[104] Chowdhury R, Webber JP, Gurney M, Mason MD, Tabi Z, Clayton A (2015). Cancer exosomes trigger mesenchymal stem cell differentiation into pro-angiogenic and pro-invasive myofibroblasts. Oncotarget.

[105] McAtee CO, Booth C, Elowsky C (2019). Prostate tumor cell exosomes containing hyaluronidase Hyal1 stimulate prostate stromal cell motility by engagement of FAK-mediated integrin signaling. Matrix Biol.

[106] Josson S, Chung LWK, Gururajan M (2015). microRNAs and prostate cancer. Adv Exp Med Biol.

[107] Fedele C, Singh A, Zerlanko BJ, Iozzo RV, Languino LR (2015). The αvβ6 integrin is transferred intercellularly via exosomes. J Biol Chem.

[108] Krishn SR, Salem I, Quaglia F (2020). The αvβ6 integrin in cancer cell-derived small extracellular vesicles enhances angiogenesis. J Extracell Vesicles.

[109] Bijnsdorp IV, Geldof AA, Lavaei M, Piersma SR, van Moorselaar RJ, Jimenez CR (2013). Exosomal ITGA3 interferes with non-cancerous prostate cell functions and is increased in urine exosomes of metastatic prostate cancer patients. J Extracell Vesicles.

[110] Honeywell DR, Cabrita MA, Zhao H, Dimitroulakos J, Addison CL (2013). miR-105 inhibits prostate tumour growth by suppressing CDK6 levels. PLoS One.

[111] Read J, Ingram A, Al Saleh HA (2017). Nuclear transportation of exogenous epidermal growth factor receptor and androgen receptor via extracellular vesicles. Eur J Cancer.

[112] Maolake A, Izumi K, Natsagdorj A (2018). Tumor necrosis factor-α induces prostate cancer cell migration in lymphatic metastasis through CCR7 upregulation. Cancer Sci.

[113] Dilsiz N (2020). Role of exosomes and exosomal microRNAs in cancer. Future Sci OA.

[114] Thakur BK, Zhang H, Becker A (2014). Double-stranded DNA in exosomes: a novel biomarker in cancer detection. Cell Res.

[115] Martin TA, Ye L, Sanders A https://www.ncbi.nlm.nih.gov/books/NBK164700/.

[116] O'Malley G, Heijltjes M, Houston AM (2016). Mesenchymal stromal cells (MSCs) and colorectal cancer: a troublesome twosome for the anti-tumour immune response?. Oncotarget.

[117] Tian W, Liu S, Li B (2019). Potential role of exosomes in cancer metastasis. Biomed Res Int.

[118] Shi A, Li J, Qiu X (2021). TGF-β loaded exosome enhances ischemic wound healing *in vitro* and *in vivo*. Theranostics.

[119] Zhang M, Wang X, Han MK, Collins JF, Merlin D (2017). Oral administration of ginger-derived nanolipids loaded with siRNA as a novel approach for efficient siRNA drug delivery to treat ulcerative colitis. Nanomedicine.

[120] Barillari G (2020). The impact of matrix metalloproteinase-9 on the sequential steps of the metastatic process. Int J Mol Sci.

[121] Gao Z, Pang B, Li J, Gao N, Fan T, Li Y (2021). Emerging role of exosomes in liquid biopsy for monitoring prostate cancer invasion and metastasis. Front Cell Dev Biol.

[122] Lorenc T, Klimczyk K, Michalczewska I, Słomka M, Kubiak-Tomaszewska G, Olejarz W (2020). Exosomes in prostate cancer diagnosis, prognosis and therapy. Int J Mol Sci.

[123] Ahearn TU, Peisch S, Pettersson A, Transdisciplinary prostate cancer partnership (topcap) (2018). expression of IGF/insulin receptor in prostate cancer tissue and progression to lethal disease. Carcinogenesis.

[124] Mancarella C, Casanova-Salas I, Calatrava A (2017). Insulin-like growth factor 1 receptor affects the survival of primary prostate cancer patients depending on TMPRSS2-ERG status. BMC Cancer.

[125] Figel S, Gelman IH (2011). Focal adhesion kinase controls prostate cancer progression via intrinsic kinase and scaffolding functions. Anticancer Agents Med Chem.

[126] Varkaris A, Katsiampoura AD, Araujo JC

[127] Khodabakhsh F, Merikhian P, Eisavand MR, Farahmand L

[128] Tang X, Zhang Q, Shi S (2010). Bisphosphonates suppress insulin-like growth factor 1-induced angiogenesis via the HIF-1alpha/VEGF signaling pathways in human breast cancer cells. Int J Cancer.

[129] Wu X, Wang J, Liang Q (2022). Recent progress on FAK inhibitors with dual targeting capabilities for cancer treatment. Biomed Pharmacother.

[130] Zhang X, Yuan X, Shi H, Wu L, Qian H, Xu W (2015). Exosomes in cancer: small particle, big player. J Hematol Oncol.

[131] Kahlert C, Kalluri R (2013). Exosomes in tumor microenvironment influence cancer progression and metastasis. J Mol Med.

[132] Crow J, Atay S, Banskota S, Artale B, Schmitt S, Godwin AK (2017). Exosomes as mediators of platinum resistance in ovarian cancer. Oncotarget.

[133] Jones E, Pu H, Kyprianou N (2009). Targeting TGF-beta in prostate cancer: therapeutic possibilities during tumor progression. Expert Opin Ther Targets.

[134] Massagué J (2012). TGFβ signalling in context. Nat Rev Mol Cell Biol.

[135] Gaglani S, Gonzalez-Kozlova E, Lundon DJ, Tewari AK, Dogra N, Kyprianou N (2021). Exosomes as a next-generation diagnostic and therapeutic tool in prostate cancer. Int J Mol Sci.

[136] Salciccia S, Capriotti AL, Laganà A (2021). Biomarkers in prostate cancer diagnosis: from current knowledge to the role of metabolomics and exosomes. Int J Mol Sci.

[137] Khan S, Jutzy JM, Valenzuela MM (2012). Plasma-derived exosomal survivin, a plausible biomarker for early detection of prostate cancer. PLoS One.

[138] Pucci M, Tavern S, Reclusa P (2017). Exosomes in semen: opportunities as a new tool in prostate cancer diagnosis. Transl Cancer Res.

[139] Duijvesz D, Luider T, Bangma CH, Jenster G (2011). Exosomes as biomarker treasure chests for prostate cancer. Eur Urol.

[140] Samsonov R, Shtam T, Burdakov V (2016). Lectin-induced agglutination method of urinary exosomes isolation followed by mi-RNA analysis: application for prostate cancer diagnostic. Prostate.

[141] Gabriel K, Ingram A, Austin R (2013). Regulation of the tumor suppressor PTEN through exosomes: a diagnostic potential for prostate cancer. PLoS One.

[142] Logozzi M, Mizzoni D, Di Raimo R (2021). Plasmatic exosome number and size distinguish prostate cancer patients from healthy individuals: a prospective clinical study. Front Oncol.

[143] Logozzi M, Mizzoni D, Capasso C (2020). Plasmatic exosomes from prostate cancer patients show increased carbonic anhydrase IX expression and activity and low pH. J Enzyme Inhib Med Chem.

[144] Li P, Yu X, Han W (2019). Ultrasensitive and reversible nanoplatform of urinary exosomes for prostate cancer diagnosis. ACS Sens.

[145] Cho S, Yang HC, Rhee WJ (2019). Simultaneous multiplexed detection of exosomal microRNAs and surface proteins for prostate cancer diagnosis. Biosens Bioelectron.

[146] Li Q, Wang Y, Ling L (2021). Rapid and specific detection nanoplatform of serum exosomes for prostate cancer diagnosis. Mikrochim Acta.

[147] Wang Y, Li Q, Shi H (2020). Microfluidic raman biochip detection of exosomes: a promising tool for prostate cancer diagnosis. Lab Chip.

[148] (2013). Andaloussi S, Lakhal S, Mäger I, Wood MJ. Exosomes for targeted siRNA delivery across biological barriers. Adv Drug Deliv Rev.

[149] Gutierrez-Millan C, Calvo Díaz C, Lanao JM, Colino CI (2021). Advances in exosomes-based drug delivery systems. Macromol Biosci.

[150] Limoni SK, Moghadam MF, Moazzeni SM, Gomari H, Salimi F (2019). Engineered exosomes for targeted transfer of siRNA to HER2 positive breast cancer cells. Appl Biochem Biotechnol.

[151] Lin D, Zhang H, Liu R (2021). iRGD-modified exosomes effectively deliver CPT1A siRNA to colon cancer cells, reversing oxaliplatin resistance by regulating fatty acid oxidation. Mol Oncol.

[152] Zhang Q, Zhang H, Ning T (2020). Exosome-delivered c-met siRNA could reverse chemoresistance to cisplatin in gastric cancer. Int J Nanomedicine.

[153] Han Q, Xie QR, Li F (2021). Targeted inhibition of SIRT6 via engineered exosomes impairs tumorigenesis and metastasis in prostate cancer. Theranostics.

[154] Krishn SR, Garcia V, Naranjo NM (2022). Small extracellular vesicle-mediated ITGB6 siRNA delivery downregulates the αVβ6 integrin and inhibits adhesion and migration of recipient prostate cancer cells. Cancer Biol Ther.

[155] Zhupanyn P, Ewe A, Büch T (2020). Extracellular vesicle (ECV)-modified polyethylenimine (PEI) complexes for enhanced siRNA delivery in vitro and in vivo. J Control Release.

[156] Kar R, Dhar R, Mukherjee S (2023). Exosome-based smart drug delivery tool for cancer theranostics. ACS Biomater Sci Eng.

[157] Pan S, Zhang Y, Huang M (2021). Urinary exosomes-based engineered nanovectors for homologously targeted chemo-chemodynamic prostate cancer therapy via abrogating EGFR/AKT/NF-kB/IkB signaling. Biomaterials.

[158] Tian J, Tai Z, Zhang W (2020). Exosomes derived from nanocomplex-loaded macrophages for targeted delivery of docetaxel and siPLK1 against castrate-resistance prostate cancer. Res Sq.

[159] Beeraka NM, Doreswamy SH, Sadhu SP (2020). The role of exosomes in stemness and neurodegenerative diseases-chemoresistant-cancer therapeutics and phytochemicals. Int J Mol Sci.

